# Comparison of ocular surface assessment and adherence between preserved and preservative-free latanoprost in glaucoma: a parallel-grouped randomized trial

**DOI:** 10.1038/s41598-021-94574-x

**Published:** 2021-07-22

**Authors:** Dai Woo Kim, Jonghoon Shin, Chang Kyu Lee, Myungjin Kim, Sohyeon Lee, Seungsoo Rho

**Affiliations:** 1 Department of Ophthalmology, Kyungpook National University School of Medicine, Daegu, Republic of Korea; 2 Department of Ophthalmology, College of Medicine, Pusan National University Yangsan Hospital, Yangsan, Republic of Korea; 3 Department of Ophthalmology, Research Institute for Convergence of Biomedical Science and Technology, Pusan National University Hospital, Yangsan, Republic of Korea; 4 Department of Ophthalmology, Ulsan University Hospital, University of Ulsan College of Medicine, Ulsan, Republic of Korea; 5 Department of Ophthalmology, CHA Bundang Medical Center, CHA University, 59 Yatap-ro, Bundang-gu, Seongnam-si, Gyeonggi-do 463-712 Republic of Korea

**Keywords:** Glaucoma, Randomized controlled trials

## Abstract

Given that nonadherence is related to subject characteristics and drug tolerance and preserved eye drops tend to be more intolerable than preservative-free ones, we conducted a phase 4, parallel-grouped, investigator-blind, active-control, randomized, multicenter study. A total of 51 patients with intraocular pressure (IOP) ≥ 15 mmHg diagnosed with open-angle glaucoma or ocular hypertension were randomly assigned to the preserved latanoprost group (n = 26) and the preservative-free latanoprost group (n = 25). The efficacy variables were corneal/conjunctival staining grade, Ocular Surface Disease Index (OSDI), adherence at 12 weeks after the first administration; corneal/conjunctival staining grade at 4 weeks; and IOP, tear break-up time (TBUT), and hyperemia score at 4 and 12 weeks. The safety variables included visual acuity and drug tolerance questionnaire results. There was no statistically significant difference in corneal/conjunctival staining grade, OSDI, or TBUT between the groups at 4 and 12 weeks. However, the adherence rate was higher and the hyperemia score was lower in the preservative-free group than in the preserved group. The severity and duration of stinging/burning sensation were lower in the preservative-free group than in the preserved group. Overall, preservative-free latanoprost showed better ocular tolerance assessed by hyperemia scores and stinging/burning symptoms following higher adherence than preserved latanoprost.

## Introduction

Glaucoma is the leading cause of global irreversible blindness, and the number of glaucoma patients aged over 40 years is estimated to increase to 111.8 million in 2040 worldwide^1^. Since the main strategy for treating glaucoma is adequate control of intraocular pressure (IOP) using antiglaucoma eye drops, physicians have been focused on how patients become more adherent to eye drop usage.

A large number of glaucoma patients experience ocular symptoms and signs upon and between instillation of antiglaucoma eye drops, which can affect the quality of life and adherence to therapy^2–4^. The ocular adverse effect appears to be the second most common reason for switching medication following low efficacy, which can lead to treatment failure and progression of visual function loss in glaucoma patients^2^.

Laboratory and clinical studies with benzalkonium chloride (BAK)-containing eye drops, an antimicrobial preservative, have shown a higher incidence of ocular signs and symptoms than BAK-free formulations^5,6^. The development of a preservative-free latanoprost eye drop is inevitable based on the known deleterious effects of BAK and the increasing interest in patients’ quality of life. A parallel-group noninferiority study comparing preserved and preservative-free latanoprost described that they have the same efficacy in terms of IOP control^7^. A study by Misiuk-Hojlo et al. showed that the rate of moderate-to-severe conjunctival hyperemia and subjective ocular symptoms were less likely to be seen during the 3-month follow-up after switching from preserved latanoprost to preservative-free latanoprost^8^. To the best of our knowledge, this is the first prospective parallel-grouped study that directly compared the ocular signs, symptoms, and adherence between the preserved and preservative-free latanoprost groups.

We assessed the corneal/conjunctival staining score, Ocular Surface Disease Index (OSDI, Allergan, Inc., Irvine, CA, USA) score, hyperemia score, tear break-up time (TBUT), adherence, and drug tolerance to compare the differences between the two groups.

## Results

**Figure 1:**
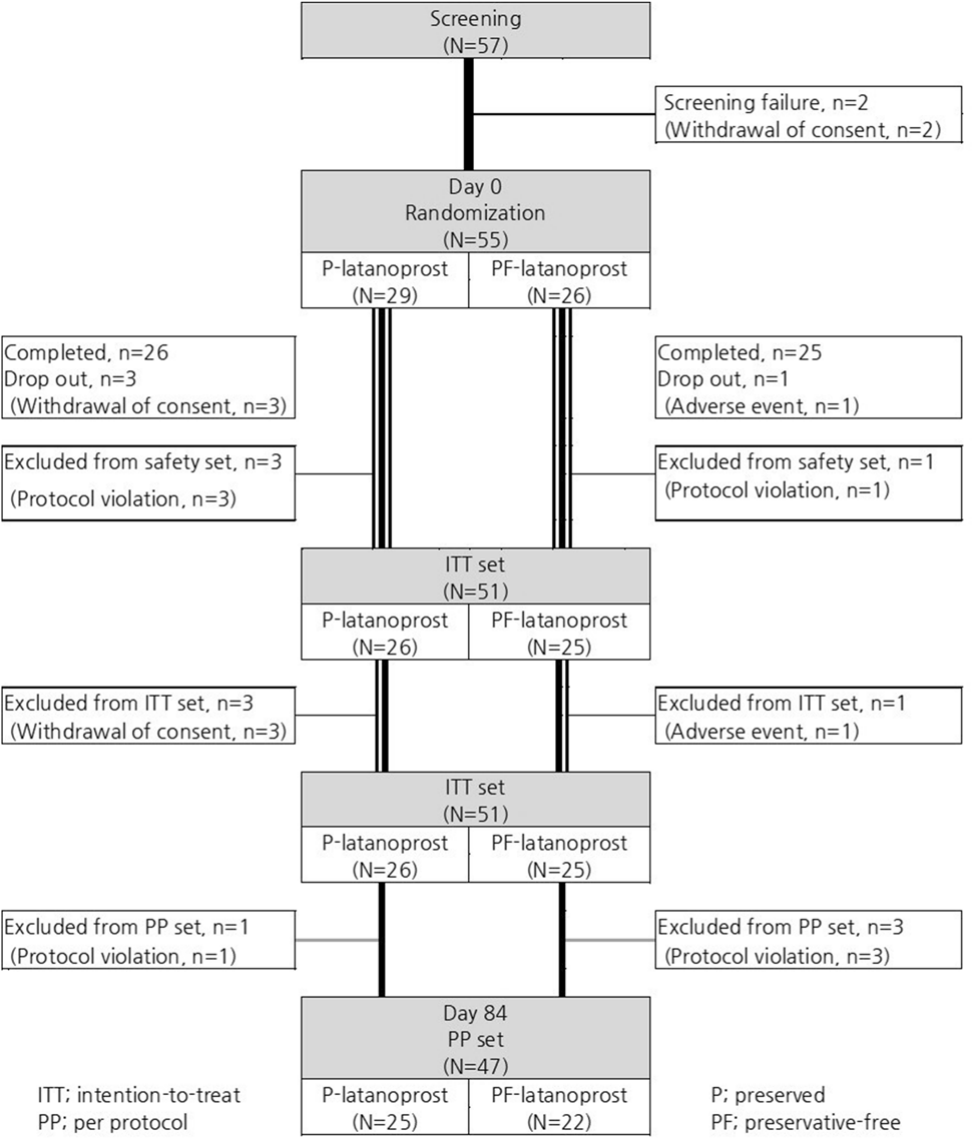
Flow chart of the subject’ enrollment.

A total of 55 (29 in the preserved group and 26 in the preservative-free group) out of 57 patients who were screened (two failed on screening due to ‘withdrawal of consent’) were finally enrolled in the study (Fig. 1). Fifty-one patients (four were excluded due to ‘withdrawal of consent’ before safety assessment at visit 3) were included in the safety set and the intention-to-treat (ITT) set (26 in the preserved group and 25 in the preservative-free group).. Four out of 51 were excluded from the per-protocol (PP) set due to protocol violation (all four of them instilled eye drops in the morning at visit 4) and a low adherence rate (two of them showed an adherence rate of < 80%). There were no differences in the demographic features between the groups (Table 1).

**Table 1:** Demographics and baseline outcome measures of the preserved and preservative-free latanoprost groups. (ITT set).

	Preserved latanoprost (n = 26)		Preservative-free latanoprost (n = 25)		*p* value
	Mean	SD	Mean	SD	
Age, years	57.85	9.28	61.36	8.57	0.167
Female, n (%)	18/26 (69.2)		14/25 (56.0)		0.393
Left eye, n (%)	14/26 (53.8)		11/25 (44.0)		0.579
SBP (mmHg)	133.69	16.00	132.88	12.48	0.841
DBP (mmHg)	78.73	11.27	78.88	8.86	0.958
PR (pulse rate, n)	72.38	11.04	73.80	8.89	0.617
Height (cm)	161.85	8.51	160.89	8.09	0.682
Weight (kg)	64.83	11.75	66.22	10.57	0.658
BMI	24.65	3.27	25.45	2.70	0.345
CCT (μm)	528.81	27.01	540.44	33.69	0.179
Baseline BCVA (decimal)	0.81	0.23	0.79	0.20	0.778
Baseline IOP (mmHg)	17.79	2.81	17.88	2.74	0.907
Baseline bulbar hyperemia	1.31	0.79	1.08	0.70	0.282
Baseline limbal hyperemia	0.81	0.85	0.84	0.94	0.898
Baseline corneal staining	0.88	0.86	0.84	1.14	0.875
Baseline conjunctival staining	5.15	4.66	5.44	4.57	0.826
Baseline TBUT	6.00	2.29	6.57	2.51	0.399
Baseline hyperemia SUM	2.12	1.40	1.92	1.50	0.632
Baseline OSDI score	17.07	12.16	17.33	14.73	0.944

*ITT, intention-to-treat; SBP, systolic blood pressure; DBP, diastolic blood pressure; PR, pulse rate; BMI, body mass index; CCT, central corneal thickness; BCVA, best-corrected visual acuity; IOP, intraocular pressure; conjunctival staining, sum of all six areas; TBUT, tear break-up time; hyperemia SUM, bulbar + limbal hyperemia score; OSDI, Ocular Surface Disease Index.

### Primary efficacy endpoint

**Figure 2:**
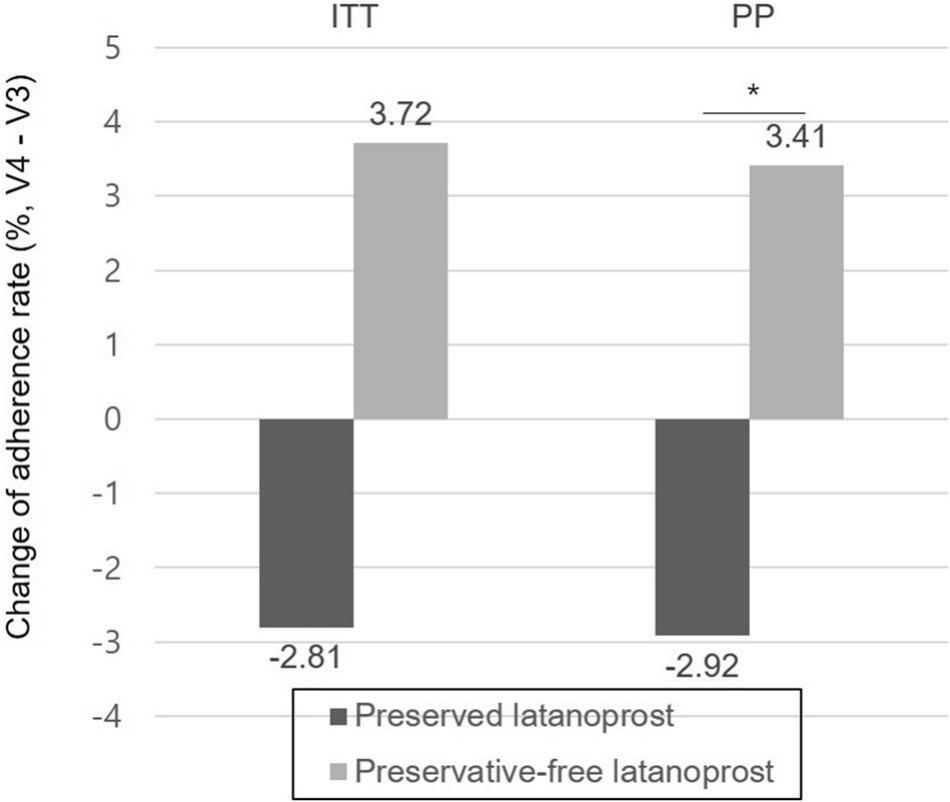
Comparison of adherence change between preserved and preservative-free latanoprost groups. (* represents statistical significance by paired t-test). ITT, intention-to-treat; PP, per-protocol.

There were no significant differences in corneal/conjunctival staining scores, or OSDI scores between the two groups in the ITT and PP sets at 12 weeks. The change in the adherence rate in the preservative-free group of the ITT set was increased at 12 weeks without statistical significance (3.72 ± 21.88%) compared with that of the preserved group (− 2.81 ± 6.66%), whereas the change showed statistical significance in the PP set (*p* = 0.019, 3.41 ± 10.82% vs. − 2.92 ± 6.77% in the preservative-free group vs. the preserved group, respectively, Fig. 2, Table 2). Analysis of covariance (ANCOVA) also supported the superiority (*p* = 0.021).

**Table 2:** Comparison of primary and secondary outcome measures between preserved and preservative-free latanoprost groups. Note that each ANCOVA test was performed within each analysis set (Preserved vs. Preservative-free; ITT set, 26 vs 25; PP set 25 vs 22).

	Preserved latanoprost		Preservative-free latanoprost		*p* value	ANCOVA *p* value
	Mean	SD	Mean	SD		
**Primary outcome measures**						
Corneal staining V4 (ITT)	0.96	0.77	0.72	0.94	0.319	0.364
Conjunctival staining V4 (ITT)	6.15	3.27	6.00	3.50	0.872	0.817
OSDI score V4 (ITT)	13.22	12.77	8.25	6.46	0.086	0.163
OSDI score V4 (PP)	13.42	13.00	9.00	6.38	0.141	0.282
Compliance V4 (ITT)	94.77	5.92	95.40	11.71	0.808	0.861
Compliance V4 (PP)	94.56	5.95	98.45	3.26	0.007	0.024
Compliance V4-V3 (ITT)	3.72	21.88	-2.81	6.66	0.152	0.492
Compliance V4-V3 (PP)	3.41	10.82	-2.92	6.77	0.019	0.021
**Secondary outcome measures**						
Corneal staining V3 (ITT)	1.00	0.98	0.80	0.71	0.409	0.399
Conjunctival staining V3 (ITT)	6.62	3.58	5.88	2.67	0.411	0.292
IOP V3 (ITT)	12.48	2.11	13.02	2.17	0.372	0.246
IOP V4 (ITT)	12.25	2.08	13.00	2.61	0.261	0.240
TBUT V3 (ITT)	6.03	2.87	5.22	1.76	0.237	0.287
TBUT V4 (ITT)	5.57	2.25	5.16	1.72	0.473	0.458
Bulbar hyperemia V3 (ITT)	1.35	0.80	1.32	0.75	0.904	0.983
Limbal hyperemia V3 (ITT)	1.04	0.72	1.08	0.81	0.847	0.917
Hyperemia SUM V3 (ITT)	2.38	1.24	2.40	1.23	0.965	0.960
Bulbar hyperemia V4 (ITT)	1.19	0.69	0.88	0.60	0.092	0.068
Limbal hyperemia V4 (ITT)	1.27	0.67	1.00	0.71	0.168	0.124
Hyperemia SUM V4 (ITT)	2.46	1.24	1.88	1.01	0.073	0.049
Bulbar hyperemia V3 (PP)	1.36	0.81	1.32	0.72	0.853	0.999
Limbal hyperemia V3 (PP)	1.04	0.74	1.09	0.81	0.822	0.968
Hyperemia SUM V3 (PP)	2.40	1.26	2.41	1.14	0.980	0.979
Bulbar hyperemia V4 (PP)	1.24	0.66	0.86	0.64	0.055	0.037
Limbal hyperemia V4 (PP)	1.28	0.68	1.05	0.72	0.257	0.200
Hyperemia SUM V4 (PP)	2.52	1.23	1.91	1.07	0.077	0.051

*V3, at visit 3; V4, at visit 4; conjunctival staining, sum of all six areas; OSDI, Ocular Surface Disease Index; IOP, intraocular pressure; TBUT, tear break-up time; hyperemia SUM, bulbar + limbal hyperemia score; ITT, intention-to-treat; PP, per protocol.

### Secondary efficacy endpoint

No statistically significant differences in corneal/conjunctival staining score at 4 weeks, changes in IOP at 4 and 12 weeks, or TBUT at 4 and 12 weeks were noted. However, the sum of bulbar and limbal hyperemia scores of the preservative-free group at 12 weeks was significantly lower than that of the preserved group in the ITT set (ANCOVA *p* = 0.049, 1.88 ± 1.01 vs. 2.46 ± 1.24, respectively). In the PP set, only the bulbar hyperemia score of the preservative-free group at 12 weeks was significantly lower than that of the preserved group (ANCOVA *p* = 0.037, 0.86 ± 0.64 vs. 1.24 ± 0.66, respectively, Table 2).

### Safety measure

The best-corrected visual acuity (BCVA) was significantly increased in the preservative-free group at 4 weeks and in both groups at 12 weeks. The severity and duration of stinging and burning sensation were significantly lower and shorter in the preservative-free group at 4 and 12 weeks than in the preserved group (ANCOVA, *p* < 0.001). Other symptoms, such as sticky sensation, itching, blurring, sandiness and grittiness, dryness, light sensitivity, and pain and soreness showed no difference between the preservative-free and preserved groups (Table 3).

**Table 3:** Comparison of drug tolerance assessed by symptoms between the preserved latanoprost group and the preservative-free latanoprost group (safety set). Note that the ANCOVA test was performed for the symptom variables and not only the duration variables when the univariate analysis showed a p-value of less than 0.05.

	Preserved latanoprost (n = 26)		Preservative-free latanoprost (n = 25)		*p* value	ANCOVA *p* value
	Mean	SD	Mean	SD		
Stinging and Burning V3	1.00	0.80	0.16	0.37	< 0.001	< 0.001
Duration	0.81	0.57	0.16	0.37	< 0.001	
Sticky sense V3	0.19	0.49	0.36	0.57	0.265	
Duration	0.23	0.59	0.36	0.57	0.429	
Itching V3	0.38	0.57	0.24	0.44	0.314	
Duration	0.42	0.64	0.24	0.44	0.239	
Blurred vision V3	0.62	0.90	0.40	0.58	0.312	
Duration	0.54	0.76	0.40	0.58	0.469	
Sandiness and Grittiness V3	0.46	0.86	0.20	0.50	0.189	
Duration	0.31	0.55	0.20	0.50	0.468	
Dryness V3	0.42	0.64	0.24	0.72	0.344	
Duration	0.38	0.57	0.20	0.58	0.257	
Light sensitivity V3	0.19	0.49	0.00	0.00	0.057	0.059
Duration	0.15	0.46	0.00	0.00	0.103	
Pain and Soreness V3	0.23	0.51	0.00	0.00	0.031	0.072
Duration	0.19	0.40	0.00	0.00	0.022	
Stinging and Burning V4	1.15	0.93	0.20	0.41	< 0.001	< 0.001
Duration	0.77	0.43	0.20	0.41	< 0.001	
Sticky sense V4	0.15	0.37	0.44	0.58	0.043	
Duration	0.15	0.37	0.40	0.50	0.052	
Itching V4	0.38	0.70	0.16	0.37	0.158	
Duration	0.31	0.47	0.16	0.37	0.220	
Blurred vision V4	0.50	0.81	0.36	0.49	0.462	
Duration	0.35	0.49	0.36	0.49	0.920	
Sandiness and Grttiness V4	0.46	0.81	0.16	0.37	0.095	
Duration	0.31	0.47	0.16	0.37	0.220	
Dryness V4	0.27	0.53	0.12	0.33	0.235	
Duration	0.23	0.43	0.12	0.33	0.307	
Light sensitivity V4	0.35	0.85	0.00	0.00	0.047	0.077
Duration	0.19	0.40	0.00	0.00	0.022	
Pain and Soreness V4	0.35	0.63	0.08	0.28	0.057	0.128
Duration	0.27	0.45	0.08	0.28	0.078	

*V3, at visit 3; V4, at visit 4.

The total incidence of adverse events (AEs) was 19.61% (10/51 patients, 13 cases). AEs were reported in 15.38% (4/26 patients, 4 cases) of the preserved group and 24% (6/25 patients, 9 cases) of the preservative-free group. The incidence of ocular surface disorders was 3.85% in the preserved group (1/26 patients, 1 case of dry eye) and 8% in the preservative-free group (2/25 patients, 1 case of dry eye, 1 case of epiphora).

## Discussions

IOP control, which can be mainly achieved by effective and regular self-administration of glaucoma eye drops, is associated with reduced progression of visual field defects according to major large-scale clinical trials^9–11^. Nonadherence to glaucoma eye drops is a significant barrier to the successful treatment of glaucoma. If we put the limited value of self-reporting data aside, PF prostaglandin analogs are relatively novel options and employing them can be one step in the delivery of successful medical therapy balancing good efficacy with tolerability and adherence^12^. A recent survey-based study reported that preservative-free eye drops may provide benefits for adherence in relation to side effects. Individuals who experienced side effects with glaucoma eye drops reported higher rates of nonadherence than those who did not (37.6% vs. 18.4%; *p* = 0.004)^11^. The self-reported nonadherence rates were 32.0%, 25.0%, and 12.5% for the preserved eye drops, combined preserved and preservative-free eye drops, and preservative-free eye drops only groups, respectively. Interestingly, even the combination of preservative-free eye drops with preserved eye drops can also decrease the rate of nonadherence. The most common side effects of glaucoma eye drops were burning sensation (49.6%), and redness (39.2%). Although the vast majority of glaucoma patients reported a high degree of satisfaction with their current eye drops with or without experiencing side effects, the nonadherence rate was significantly lower in the no side effects group, indicating that the avoidance of even minor side effects can be beneficial to patients.

Given the differences between the unit-dose pipettes and the multiple-use bottles, one can assume that the type of eye drop container might affect patient adherence. Na et al. reported that the proportions of proper consumers of glaucoma eye drops were higher in the unit groups than in the bottle groups. In their study, three quarters or more of subjects in the unit groups were in the narrower range of proper consumption, suggesting that prescribing eye drops with unit-dose pipettes could lead to more consistent consumption of medication^13^. Proper consumption is also important for the study design itself since the change of adherence, side effects, and drug tolerance can all be affected by it. We assume that the lack of statistical significance in the ITT set of our study despite the positive change in adherence rate (Fig. 2) was due to outliers influenced by improper consumers (protocol violations) whereas statistical significance was seen in the PP set. Due to the limitation of our study design, we were not able to determine the extent to which the container affected adherence. Further studies are needed to verify this hypothesis.

A number of studies have reported that chronic application of preserved eye drops induces significant detrimental effects on the ocular surface. BAK, the most commonly used preservative in eye drops to date, was originally introduced by Gustav Raupenstrauch as an antiseptic disinfectant in Germany for the control of the Cholera pandemic in 1889. BAK is highly toxic to fish (LC50 = 280 μg a.i./L), moderately toxic to birds (LD50 = 136 mg/kg bw), and slightly toxic to mammals (LD50 = 430 mg/kg bw) on an acute exposure basis^14^. BAK disrupts the lipid layer of the cell membrane of pathogens, such as *Vibrio cholerae* whereas it also exerts harmful effects on the ocular surface when added to eye drops by destabilizing the tear film, causing inflammation, squamous metaplasia, and fibrotic changes in the conjunctiva^5,15–18^.

Despite the studies supporting the toxic effect of BAK on the ocular surface, the beneficial aspect of using BAK in eye drops is still debated. The assumption that BAK can improve the pharmacologic effects of antihypertensive agents as a penetration enhancer through the cornea has been contradicted by a considerable number of clinical studies^19,20^. BAK-free travoprost showed a similar IOP-lowering effect as BAK-containing travoprost^21^. A comparative study reported that there was no significant difference in the tafluprost concentration in rabbit aqueous humor between BAK-free and BAK-containing eye drops^22^. Among the preserved glaucoma eye drops, latanoprost and its generics contain higher concentrations of BAK (0.02%) than all the other monotherapy eye drops, which draws the conceptualization of this study.

In our study, there were no significant differences in corneal/conjunctival staining scores, or OSDI scores between the preserved and non-preserved groups at all measurements. However, this finding does not necessarily mean that BAK is not toxic to the ocular surface; preferably, it could mean that the acute detrimental effect of BAK does not last long if the exposure time is a fairly short term. A recent study described that preserved latanoprost caused a significantly acute decrease in transepithelial electric resistance (TER) measurement of the corneal epithelium at 1 min after the first instillation^23^. Surprisingly, the decrease disappeared at 24 h as well as at 1 week after once-daily application of the preserved latanoprost, and these findings were confirmed by scanning electron microscopy analyses. TER reflects the barrier function of the corneal epithelium; therefore, corneal TER is considered suitable for the quantitative assessment of corneal permeability and irritancy. This regenerative power seems to be a repetitive process during the daily exposures to BAK and it might be the reason why the ocular surface findings and OSDI scores failed to show significant differences since the follow-up period in this study was 1 month and 3 months from the first visit.

Alternatively, the detrimental effect on the corneal surface might have been more significant if the follow-up period was sufficiently long to result in chronic changes, given that BAK toxicity is cumulative. Moreover, the majority of the subjects in our study (except four subjects in the PP set) were naive to glaucoma treatment, which might explain why their ocular surface was less vulnerable than expected. The benefit will be far greater when multiple medications are used^24^. This needs to be addressed in the future as latanoprost eye drops are most commonly prescribed eye drops in many countries, including South Korea^25^. In the same vein, a systematic review is not enough to explain a global switch from preserved eye drops to PF eye drops, although a meta-analysis has shown that there are no clinically significant differences in ocular hyperemia or tear break-up time^26^. The lack of difference may be due to variations in design, study quality, and outcome definition.

This study used a parallel-group design instead of a crossover design. The crossover design requires a much smaller number of patients for similar statistical power because subjects act as their own controls, resulting in a lower financial cost and exposure of fewer patients to each agent. In contrast, there is a theoretical risk that the effects of the first intervention might carry over into the second intervention, possibly confounding the detection of effects^27^. The parallel-group design is more versatile in a study with relatively stable disease if a multicenter approach is possible. Therefore, we conducted a parallel study that was more beneficial for investigating the effect of eye drops on ocular surface status. In addition, a preemptive validation of ocular assessment was performed to minimize potential bias between the examiners.

In conclusion, preservative-free latanoprost shows better ocular tolerance assessed by hyperemia scores and stinging and burning symptoms following higher adherence than preserved latanoprost in open-angle glaucoma or ocular hypertensive eyes with unfailingly comparable IOP-lowering effects. Close examination of the detrimental effect of BAK on the ocular surface should be verified by a long-term study design, since glaucoma medications are normally considered a chronic option.

## Methods

### Subjects enrollment and study design

**Figure 3:**
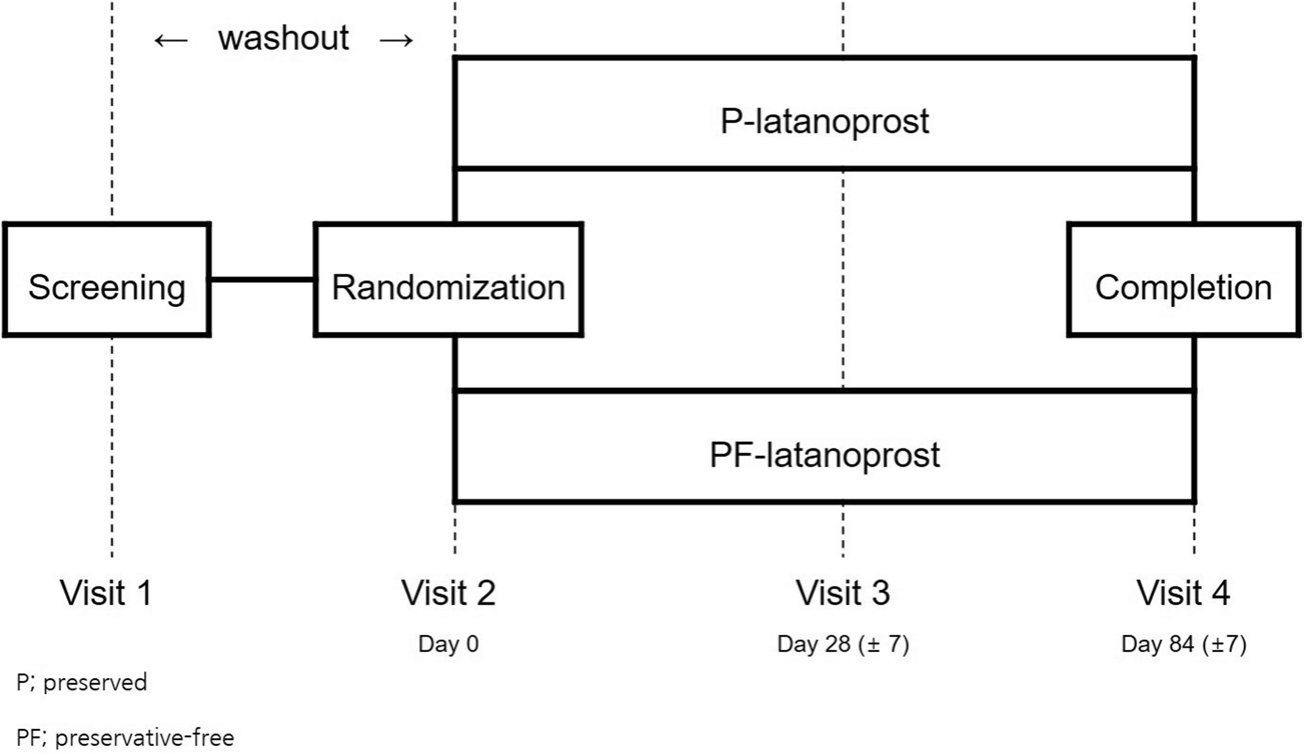
Follow-up schedule of the study.

This study was a parallel-grouped, investigator-blind, active-control, randomized, multicenter (four institutions), and phase 4 clinical trial approved by the Institutional Review Board of CHA Bundang Medical Center on 20/02/2019, which fully adhered to the Declaration of Helsinki. The study was registered on clinicaltrials.gov on 06/02/2021 and posted on 08/02/2021 as NCT04743622. All subjects provided informed consent before the screening. Patients were randomized into two groups; a preservative-free latanoprost (Monoprost, Samil Pharmaceutical Company Ltd., Seoul, South Korea) group and preserved latanoprost (Xalatan, Pfizer Inc., Puurs, Belgium) group according to the interactive web-based randomization system (IWRS, TnW software Ltd., Seoul, South Korea) running 24 h a day during the whole study period. All the variables were uploaded to web-based e-CRF (case report form, http://www.ecrf.kr, ver 1.0, TnW software Ltd., Seoul, South Korea) software. All investigators were blinded throughout the whole study period due to the same external package for the investigation product, but the participants could know after receiving and unpacking the contents. Patients were instructed to instill eye drops once daily at 9 PM (± 1 h) from day 0 and were instructed to visit the clinic at 4 and 12 weeks (Fig. 3).

Patients aged 19 years or older with open-angle glaucoma or ocular hypertension were enrolled in four different institutions from April 2019 to June 2020. Glaucomatous changes in the eye were confirmed by reproducible glaucomatous visual field defects corresponding to typical optic disc/retinal nerve fiber layer changes by glaucoma specialists. All subjects underwent a full ophthalmologic examination including BCVA, Goldmann applanation tonometry by masked examiner(s) in each institution, central corneal thickness (CCT), gonioscopy, visual field test using a Humphrey Field Analyzer (Carl Zeiss Meditec, Dublin, CA), fundus photography, red-free photography, and spectral-domain optical coherence tomography.

The Inclusion criterion was an IOP of 15 ~ 40 mmHg in at least one eye at the screening visit after the proper period for washout (e.g., cholinergic eye drops and carbonic anhydrase inhibitors for 5 days and all other glaucoma eye drops for 4 weeks). Patients were excluded if their BCVA was worse than 20/80; if their CCT was not within 470–591 μm; if they had any eye disease (e.g., ischemic optic neuropathy, proliferative diabetic retinopathy, age-related macular degeneration, etc.) that could affect the visual field results significantly, if they had active ocular inflammatory conditions; if they had lacrimal punctal occlusion procedures in the past 3 months; if they needed to use eye drops for severe dry eye disease with hyaluronic acid, cyclosporine, or diquafosol; or if they were pregnant or currently nursing.

### Outcome measures

According to the aim of this study, the primary endpoints were the difference in the corneal staining grade (assessed by the Oxford grading system; 0–5), conjunctival staining grade (assessed by the NEI scale; the conjunctiva was divided into 6 areas; 0–3 for each area), OSDI score, and adherence change at 12 weeks between the preserved and preservative-free groups. Adherence was assessed using a self-report sheet, which was collected at 4 and 12 weeks (0–100%)^28–30^. The secondary endpoints were the difference in corneal staining grade, conjunctival staining grade, OSDI score, TBUT, and hyperemia score (assessed using the Efron grading scale; 0–4) at 4 weeks and the difference in IOP, TBUT, and hyperemia score at 12 weeks between the two groups^31,32^.

The safety outcome measures included BCVA, AEs, and drug tolerance. Drug tolerance data were acquired using a drug tolerance questionnaire sheet to collect the frequency and severity of the symptoms that occurred after the instillation of eye drops, such as stinging/burning, sticky sensation, itching, blurring, sandiness/grittiness, dryness, light sensitivity, and pain/soreness. The level of each symptom was instructed to range from 0 (none) to 3 (severe, which immensely interferes with the subject’s daily life). The duration of each symptom was graded as 0 (prompt: < 5 min) or 1 (continuous: ≥ 5 min).

### Statistical analysis

This study aimed to evaluate the superiority of preservative-free latanoprost over preserved latanoprost in terms of ocular surface conditions such as corneal/conjunctival staining, and hyperemia score. The superiority test was based on the 95% confidence interval of the difference between the two groups using an independent *t *test. Although there was no equally designed study similar to ours, superiority was concluded if the difference in the hyperemia score was 0.82 or more according to a study that evaluated the difference in eye redness before and after switching the subjects’ eye drop from preserved latanoprost to preservative-free latanoprost^8^. Given that a standard deviation of 1.0 for the hyperemia score was proposed assuming a dropout rate of 20%, a total of 62 patients (31 in each group) should be enrolled to provide 80% power for the superiority calculation. To maximize the accuracy of the assessment among all investigators, a blinded person created a validation image set of conjunctival hyperemia, which was used to check the agreement between each investigator. The Kendall’s taus of bulbar and limbal hyperemia scores between each assessor were 0.563–0.939 (*p* < 0.019) and 0.662–0.977 (*p* < 0.026), generally representing a moderate-to-high correlation.

Comparisons of the primary/secondary efficacy and safety endpoints were performed using ANCOVA to explore any possible effect of the dropout pattern on the analysis after searching for potentially significant variables using an independent t-test. Discrete or categorical variables were compared using chi-square analysis. A paired t-test was used to compare the V4–V3 change in the adherence rate between the two groups. All analyses were performed using PASW software (version 18.0; SPSS, Inc., Chicago, IL, USA). Statistical significance was set at *p* < 0.05.

Efficacy and safety assessments were performed using ITT and PP. Only patients who did not violate the protocol with an adherence rate of more than 80% were included in the PP set.

## Data Availability

All relevant data are within the paper.
